# Global health preparedness for biosecurity threats: a review of emerging technologies and solutions

**DOI:** 10.3389/fpubh.2026.1786846

**Published:** 2026-06-05

**Authors:** Omotade Adebimpe Ijarotimi, Adeyemi Akeem Amuda, Christopher Oluwatosin Omokanye, Mariam Amuda

**Affiliations:** 1 Department of Obstetrics, Gynaecology and Perinatology, Faculty of Clinical Sciences, College of Health Sciences, Obafemi Awolowo University, Ile-Ife, Nigeria; 2 Department of Obstetrics, Gynaecology and Perinatology, Obafemi Awolowo University Teaching Hospitals Complex, Ile-Ife, Nigeria; 3 Department of Community Health, Obafemi Awolowo University Teaching Hospitals Complex, Ile-Ife, Nigeria

**Keywords:** bioterrorism, biosafety, global health, emerging technologies, preparedness

## Abstract

Biosecurity threats are issues of public health importance because of their implications for global health security. Although some strategies have been implemented by global organizations to mitigate risks from natural, accidental, or deliberate biological threats, empirical data on global health preparedness for biosecurity threats generally are still evolving. Hence, this study aimed to synthesize existing evidence of the role of emerging technologies in enhancing global health preparedness for biosecurity threats. Using a PRISMA-guided protocol, we conducted a systematic review of (peer-reviewed) published and grey articles regarding biosecurity preparedness and emerging technologies in PubMed, Google Scholar, and EBSCOHOST from 2015 to 2026. Extracted data were used for narrative synthesis. Most relevant and representative 51 out of a total of 349 identified papers were selected and reviewed for this study. Key technologies identified include artificial intelligence, genomic surveillance, synthetic biology, biosensors, robotics, and digital health platforms. These technologies improve outbreak detection, surveillance, and response coordination. However, challenges include dual-use risks, ethical concerns, inequitable access, and regulatory gaps. We conclude that emerging technologies offer great prospects for enhancing global health preparedness but require robust governance frameworks, equitable access, and interdisciplinary collaboration to mitigate risks and maximize benefits. A multidisciplinary approach involving synergy between relevant stakeholders is required to combat biosecurity threats, as the world has become a global village through advances in technology.

## Introduction

Biosecurity threats are increasingly recognized as global concerns due to their potentially catastrophic impacts on human health and well-being worldwide. These threats originate from various sources, including naturally occurring pandemics, accidental laboratory leaks of virulent organisms, and bioterrorism (1). They can contribute to and worsen the rising incidents of emerging and re-emerging infectious diseases and antimicrobial resistance. In recent years, the occurrence of climate change and globalization of trade and commerce have increased human mobility and heightened these threats because of their potential to facilitate the rapid transmission of pathogens and alter ecological dynamics (2). These, in turn, can lead to the emergence of new infectious agents.

There have been many international efforts to strengthen global biosecurity and safeguard the world from the threats posed by infectious diseases. Collaborative global partnerships have led to the development of several policies and frameworks, such as the World Health Organization’s International Health Regulations (IHR, 2005) (3), the ‘One Health’ approach (4, 5), and the Global Health Security Agenda (GHSA) (6). Despite these efforts, the emergence of the COVID-19 pandemic highlighted the vulnerabilities in existing global health systems and underscored the need for innovative solutions. In this context, emerging technologies offer promising prospects for transforming biosecurity by providing new tools for the prevention, detection, and response to biosecurity threats (4, 7–9). To the best of the researchers’ knowledge, limited studies had comprehensively synthesized both published and grey literature regarding global health preparedness for biosecurity threats within the context of emerging technologies and solutions. Therefore, this article highlighted new technological tools in the global health preparedness arsenal and possible challenges to their use.

## Methodology

The authors followed the Preferred Reporting Items for Systematic Reviews and Meta-Analyses (PRISMA 2020) guidelines for this review (10). We searched PubMed, Google Scholar, and EBSCOHOST, with the keywords or phrases which included: “Biosecurity threats,” “Global health,” “Preparedness”, and “Solutions” in all fields. Appropriate Boolean operators were used to widen the scope of the search.

Our review included peer-reviewed and grey papers published from 2015 to 2026, which focused on biosecurity threats and preparedness technologies. We excluded non-English articles, those that had editorials without substance analysis, and those considered less relevant to public health interventions. Two of the authors independently screened titles, abstracts, and full texts. The authors resolved disagreements in selecting the final articles via discussion and consensus. Data extracted from the articles included study characteristics, type of technology, emerging solutions, and key findings.

Furthermore, we did quality assessment of the selected articles by using the adapted Critical Appraisal Skills Programme (CASP) and Joanna Briggs Institute (JBI) tools to assess study design rigour, methodological transparency, and relevance to research objectives (11).

## Results

### PRISMA flow diagram

**Figure 1:**
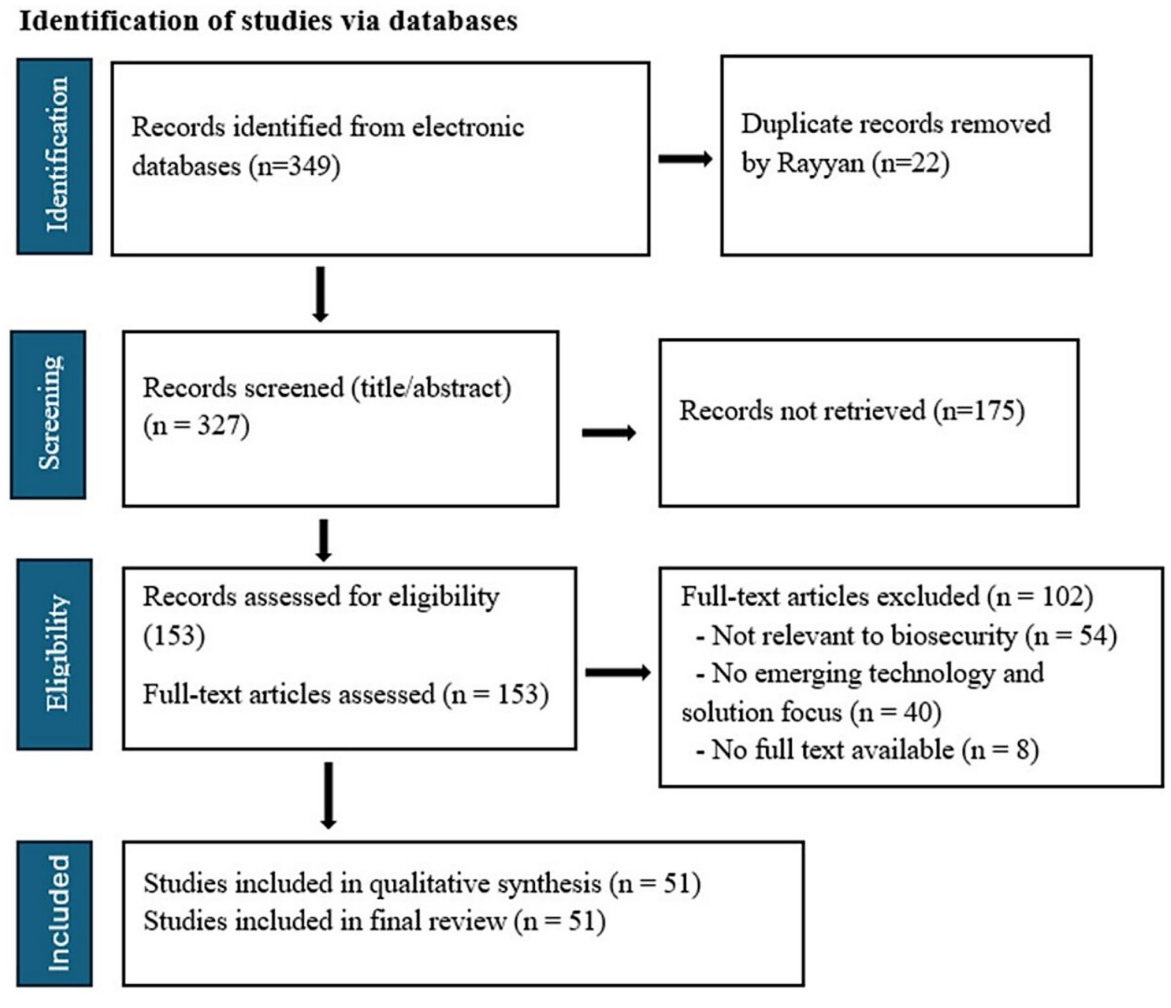
Search and selection flow diagram.

Search and selection flow diagram shown in Figure 1.

### Characteristics of included studies

The characteristics of the included literature used for the review comprised 9 internet materials and web-based resources [reference no. (1, 5, 6, 20, 21, 23, 24, 32, 51)], of which 3 were policy/white papers and organizational reports [reference no. (1, 6, 51)]. A total of 38 research articles were included [reference no. (2, 3, 4, 7, 9, 12, 13, 14, 15, 17, 18, 19, 22, 25, 26, 27, 28, 29, 31, 33, 34, 35, 36, 37, 38, 39, 40, 41, 42, 43, 44, 45, 46, 47, 48, 49, 52, 53)], while 4 were review articles [reference no. (8, 16, 30, 50)]. In addition, 2 articles were utilized as methodological guideline references [reference no. (10, 11)]. In terms of geographical distribution, the papers were from high-income and low- and middle-income countries.

### Nature and scope of biosecurity threats

Emergence of biosecurity threats can be new, re-emerging, or engineered (12–14). Examples of such threats include emerging infectious diseases, antimicrobial resistance, laboratory accidents, and bioterrorism. These threats, if not curtailed, have existential implications for human race (15). Recent outbreaks such as Coronavirus Disease 2019 (COVID-19), Lassa fever, and mpox demonstrate the persistent and evolving nature of global biological risks (12, 16). These threats are further compounded by concerns relating biological weapons, antimicrobial resistance, laboratory security and cybersecurity vulnerabilities within healthcare systems (2, 8, 12).

### Key thematic findings of emerging technologies

#### Artificial intelligence (AI) and digital surveillance

The review indicates that use of AI can be used to predict, do real-time surveillance and make responsive and timely decisions on disease outbreak. Enhances outbreak prediction, real-time surveillance, and decision-making (8, 17–19). Similarly, machine learning models can be used for timely interventions through early detection and epidemic forecasting (20, 21).

#### Genomic surveillance and diagnostics

Use of genomic technologies are increasing being used for rapid pathogen identification and tracking of mutations (13, 22, 23). Field surveillance and outbreak containment of diseases can be done using portable sequencing tools (18).

#### Synthetic biology and vaccine development

Synthetic biology has the potential for rapid development of vaccines and therapeutics, particularly mRNA platforms (14, 17, 18).

#### Biosensors, robotics, and nanotechnology

Biosensors and robotics enable early detection and frontline response activities (18, 24). Similarly, nanotechnology can improve diagnostics capability of epidemiological tools (25, 26).

#### Integrated surveillance and one health approaches

Use of integrated systems and one health approaches can enhance real-time monitoring across human, animal, and environmental health fields (4, 17, 27, 28).

#### Governance and ethical challenges to emerging solutions

There must be tight regulatory oversights to prevent and/ or mitigate the potential hazards risks associated with the emerging technologies and solutions. As widely reported across the studies, concerns about emerging technologies to biosecurity threats are dual-use risks (14, 27, 29), data privacy and security (8, 15, 30, 31), and inequitable access (17, 19). Hence, there are needs for the development and implementation of effective governance frameworks to ensure responsible use and equitable distribution of emerging technologies to biosecurity threats.

## Discussion

**Figure 2:**
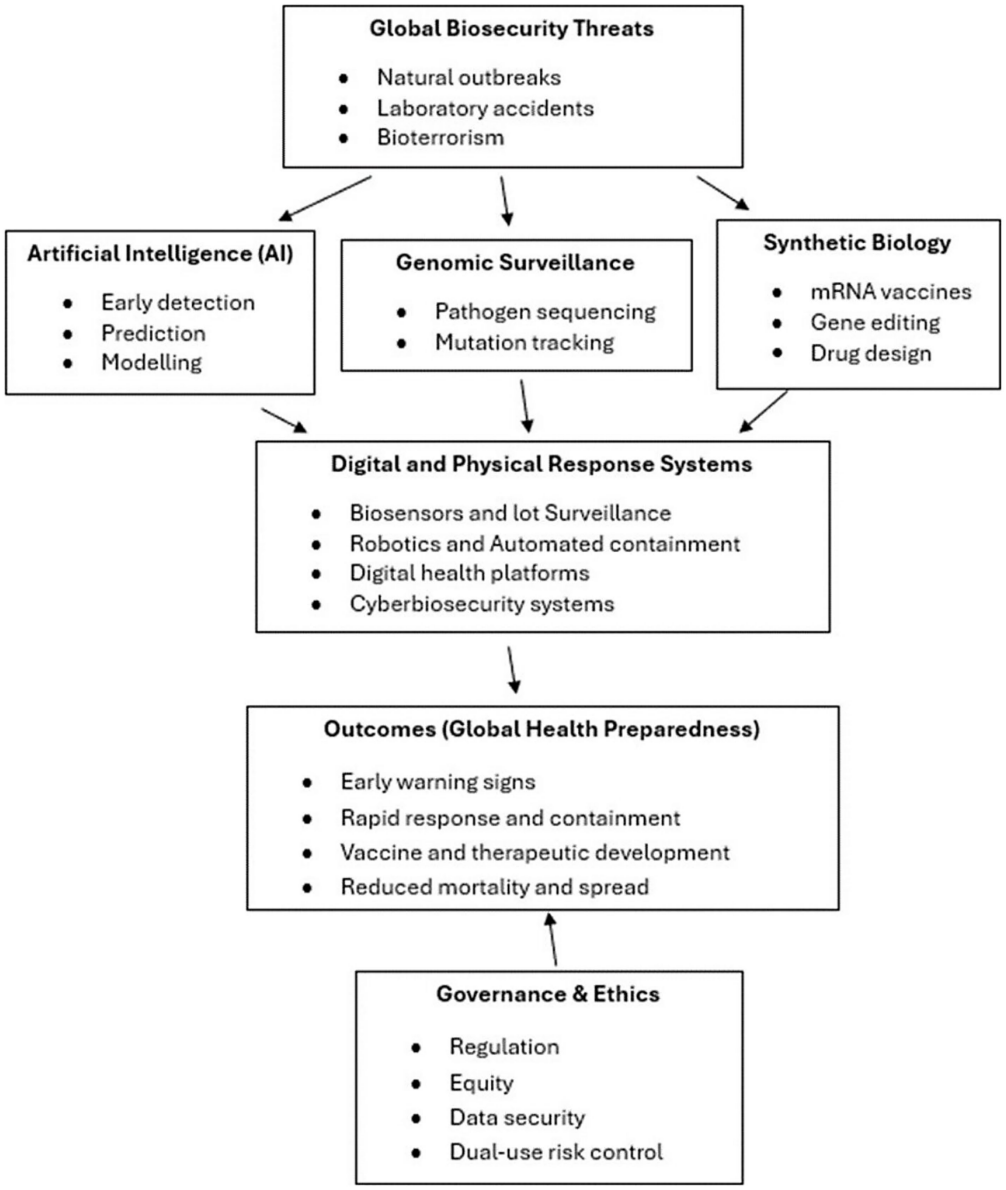
Integrated role of emerging technologies in global health preparedness for biosecurity threats.

The schematic (Figure 2) is adapted from integrated models of biosecurity systems combining AI, surveillance, genomics, and One Health approaches (32). It illustrates the integrated framework of emerging technologies in global biosecurity preparedness, highlighting how artificial intelligence, genomic surveillance, synthetic biology, and digital health systems interact to enhance early detection, response, and mitigation of biosecurity threats across global regions. Furthermore, the schematic shows the reciprocal influence of ethical and governance concerns on the applicability and effectiveness of these emerging technologies and interventions.

Biosecurity threats may have adverse effects on the health of individuals and communities. Gathering evidence across studies from all continents of the world, this review emphasized the transformative role of emerging technologies in strengthening global preparedness and response to biosecurity threats. Emerging technologies to biosecurity threats in the review were artificial intelligence and digital surveillance, genomic surveillance and diagnostics, synthetic biology and vaccine development, biosensors, robotics, and nanotechnology, integrated surveillance and one health approaches. In addition, the review reported the potential challenges associated with emerging solutions.

Artificial intelligence and digital surveillance systems have been used for outbreak prediction, real-time monitoring, and rapid decision-making in many parts of the world. For instance, data revealed that AI-supported epidemiological models were used for prediction and early detection of COVID-19 transmission in China (33, 34). Evidence from South Korea also suggests that integrating machine language into digital surveillance systems facilitated rapid contact tracing and outbreak containment of COVID-19 (35). Similarly, the use of digital surveillance systems like the Surveillance Outbreak Response Management and Analysis Systems (SORMAS) tool has assisted in efficient and effective case management of Ebola Virus Disease (EVD) and Lassa fever via early detection and timely responses in Nigeria (9, 36, 37). Across different parts of the South America and Australia, digital platforms have also been used to strengthen public health measures to contain biosecurity threats (38, 39).

Genomic surveillance and diagnostics are now essential tools for rapid pathogen identification and tracking of mutations. In Africa, Nigeria deployed genomic technologies to expand sequencing capacity during the COVID-19 pandemic (40, 41), while its use in South Africa led to the early identification of SARS-CoV-2 variants of concern (7). China and India successfully used the emerging solutions to rapidly detect viral mutations and transmission pathways of outbreak infections (29, 42). Furthermore, genomic data sharing through platforms such as Global Initiative on Sharing All Influenza Data (GISAID) have enabled real-time global tracking of pathogen evolution in North America and Europe (43, 44). Similarly, Brazil in South America implemented genomic epidemiology approaches despite resource constraints (45).

Among the most popular technologies that have been deployed to combat biosecurity threats is synthetic biology. This innovation has revolutionized vaccine and therapeutic development, particularly mRNA platforms. Synthetic biology approaches led to rapid development of COVID-9 vaccines in North America and Europe (46, 47). Similarly, advanced recombinant and viral vector vaccine technologies have been deployed to support pandemic response in China and India (29, 48). There are ongoing efforts among African countries to produce local vaccines using emerging technologies (18). Countries such as Australia have deployed synthetic biology technology-adapted vaccines for trials and manufacturing partnerships (48).

Biosensors, robotics, and nanotechnology have been deployed to detect and respond in disease outbreaks in many countries. For instance, mobile biosensor-based diagnostic tools have been used for diseases such as malaria and tuberculosis, improving access to rapid testing in Africa. At the peak of COVID-19 infection, China deployed robotic technologies for disinfection and patient care, while wearable biosensors enabled continuous health monitoring (34). In North America and Europe, nanotechnology-based diagnostic platforms have been used to improve diagnostic tool sensitivity as well as reduced detection time (49). Similarly, South American countries have explored biosensors for monitoring vector-borne diseases (18).

Another emerging technologies and solutions to the issue of biosecurity threats are integrated surveillance and one health approaches. These approaches are used for effectively addressing zoonotic and environmental health threats. A systematic review across countries in sub-Saharan Africa showed the successful implementations of One Health frameworks in responding outbreaks of Ebola, Rift Valley fever and viral zoonotic diseases (16). Similarly, using an integrated approach using human–animal surveillance systems to manage avian influenza and rabies improve timeliness, sensitivity, and response coordination, as indicated in a systematic review in Asian settings (50).

Although the emerging technologies and solutions to global biosecurity threats are promising, evidence indicates that their deployment on a large scale should be done in a cautious manner because of governance and ethical challenges. For instance, there are potential privacy and security issues related to AI-surveillance systems in Africa, Asia, and Europe (29, 51, 52). In addition, synthetic biology technologies have been documented to be associated with dual-use risks in North America and Europe (14, 53). Furthermore, there are also concerns about the challenge of equitable access to the emerging technologies in LMICs, especially SSA (18, 30).

## Conclusion

Evidence from all continents demonstrates that emerging technologies play a critical role in strengthening global biosecurity preparedness. The integrated framework, in particular, emphasizes the synergistic interaction of AI, genomic surveillance, synthetic biology, and digital health systems in enhancing early detection, response, and mitigation of biosecurity threats. However, maximizing their impact requires coordinated global action, strong governance, and equitable access to ensure that all regions benefit from these innovations.
